# Tetralogy of Fallot with rheumatic mitral stenosis: A case report

**DOI:** 10.1186/1752-1947-2-127

**Published:** 2008-04-28

**Authors:** Cheemalapati Sai Krishna, Gangireddy Venkateswara Reddy, Mohan Debta, Nanda Kishore Panigrahi

**Affiliations:** 1 Department of Cardio-Thoracic Surgery, Apollo Heart Institute, Visakhapatnam, Andhra Pradesh, India; 2 Department of Cardiology, King George Hospital, Visakhapatnam, Andhra Pradesh, India; 3 Department of Cardiology, Apollo Heart Institute, Visakhapatnam, Andhra Pradesh, India

## Abstract

**Introduction:**

Rheumatic and congenital heart diseases account for the majority of hospital admissions for cardiac patients in India. Tetralogy of Fallot is the most common congenital heart disease with survival to adulthood. Infective endocarditis accounts for 4% of admissions to a specialized unit for adult patients with a congenital heart lesion. This report is unique in that a severe stenotic lesion of the mitral valve, probably of rheumatic aetiology, was noted in an adult male with Tetralogy of Fallot.

**Case presentation:**

An unusual association of rheumatic mitral stenosis in an adult Indian male patient aged 35 years with Tetralogy of Fallot and subacute bacterial endocarditis of the aortic valve is presented.

**Conclusion:**

In this case report the diagnostic implications, hemodynamic and therapeutic consequences of mitral stenosis in Tetralogy of Fallot are discussed. In addition, the morbidity and mortality of infective endocarditis in adult patients with congenital heart disease are summarized. The risk of a coincident rheumatic process in patients with congenital heart disease is highlighted and the need for careful attention to this possibility during primary and follow-up evaluation of such patients emphasized.

## Introduction

Rheumatic and congenital heart diseases account for about 65% of total cardiac admissions in India and the prevalence rate of rheumatic heart disease is reported as 0.5 to 0.67 per 1000 [1]. Coexistent rheumatic disease in patients with congenital heart defects is known to occur [2,3]. We discuss an interesting association of rheumatic mitral stenosis in an adult with tetralogy of Fallot (TF) complicated by infective endocarditis of the aortic valve.

TF remains the most common type of congenital heart lesion seen beyond infancy and childhood with about 5% of patients surviving to the age of 40 years. Aortic regurgitation may occur in affected patients in or beyond the second decade of life, and this may predispose to infective endocarditis [4]. Mitral inflow obstruction in congenital heart disease may be due to a supramitral ring, congenital or acquired valvular stenosis and parachute mitral valve, all of which result in an elevated transmitral gradient – the hemodynamic hallmark of mitral stenosis [5]. In TF, an increasing severity of mitral stenosis potentiates the passive pulmonary venous congestion which may aggravate the hypoxemia.

Atrial fibrillation is an uncommon rhythm disturbance in TF. However, mitral stenosis may predispose the patient to atrial fibrillation, with disastrous hemodynamic consequences. The possibility of left atrial clot formation and embolic stroke may add to central nervous system complications such as cerebral thromboses and abscesses that occur as a result of a widespread tendency to spontaneous arterial thrombosis secondary to a hypercoagulable state in adult TF [4].

The low pulmonary blood flow situation in TF, with a consequent decrease in the left atrial flow, tends to result in a small left atrium and left ventricle. Mitral stenosis results in a further reduction of the left ventricular end diastolic volume, which is an important risk factor for early death after repair [4]. Myocardial damage secondary to chronic pre-operative hypoxia may adversely influence postoperative left ventricular function in older patients. Valvular calcification and sub-valvular fusion may preclude chordal preservation during surgical intervention, adding to morbidity and mortality following surgical repair.

Infective endocarditis accounts for 4% of admissions to a specialized unit for adult patients with a congenital heart lesion. Predisposing events identified include a dental procedure, systemic sepsis or prior cardiac surgery [6]. The aortic and pulmonary valves in TF are prone to infective endocarditis. The mitral valve may be an additional site for endocarditis in TF with mitral stenosis. The morbidity of infective endocarditis in this subset of patients includes recurrence (7.7%), systemic septic embolization (30.8%) and a high re-operation rate during or after the episode (67.3%). Hospital mortality is reported to be around 1.9% and late mortality 7.7% [7].

## Case presentation

A male aged 35 years with a history of cyanosis from early childhood was referred for evaluation of low grade fever and worsening breathlessness on exertion. There was no history of a recent dental or surgical procedure. General physical examination revealed a moderately nourished individual with central cyanosis and grade IV clubbing. The jugular venous pulse was elevated. The pulse was regular with a rate of 100 beats per minute and a collapsing character. The blood pressure was 120/60 mmHg. On precordial examination the first heart sound was palpable. Auscultation revealed a loud first heart sound, single second heart sound and an apical opening snap. Additional findings included ejection systolic and early diastolic murmurs at the left sternal border and a mid-diastolic murmur with pre-systolic accentuation at the apex. The lungs were clear. Abdominal examination revealed a tender enlarged liver. There was no enlarged spleen. Pulse oximetry showed a room air oxygen saturation of 78%. Chest X-ray revealed enlarged heart, pulmonary oligemia and no evidence of pulmonary venous hypertension. Electrocardiogram revealed sinus rhythm, normal PR interval, right axis deviation, left atrial enlargement and right ventricular hypertrophy.

**Figure 1:**
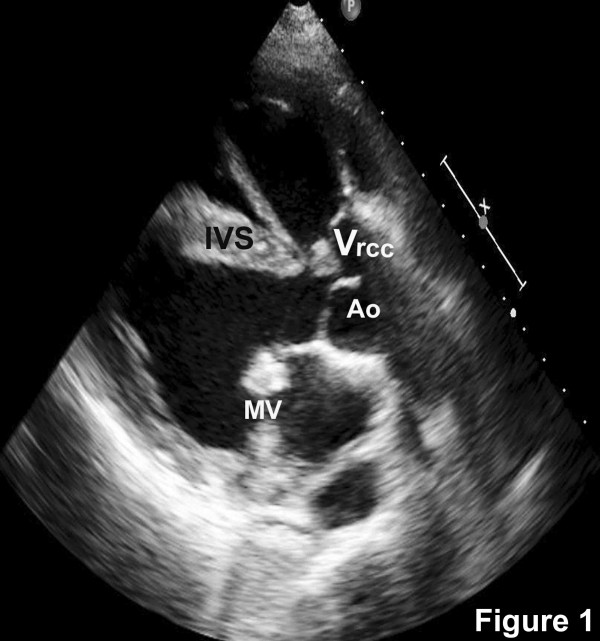
Parasternal long axis view showing the malaligned ventricular septal defect, aortic override (Ao), vegetation on the right coronary cusp (V_rcc_) and thickened mitral valve (M).

**Figure 2:**
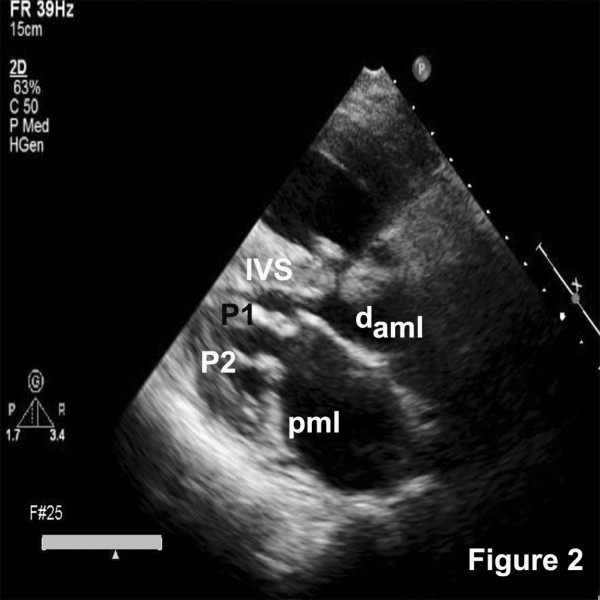
Parasternal long axis view demonstrating the papillary muscles (1 and 2), doming of the anterior mitral leaflet (d_aml_) and fixed posterior mitral leaflet (pml); IVS, interventricular septum; LA, left atrium.

**Figure 3:**
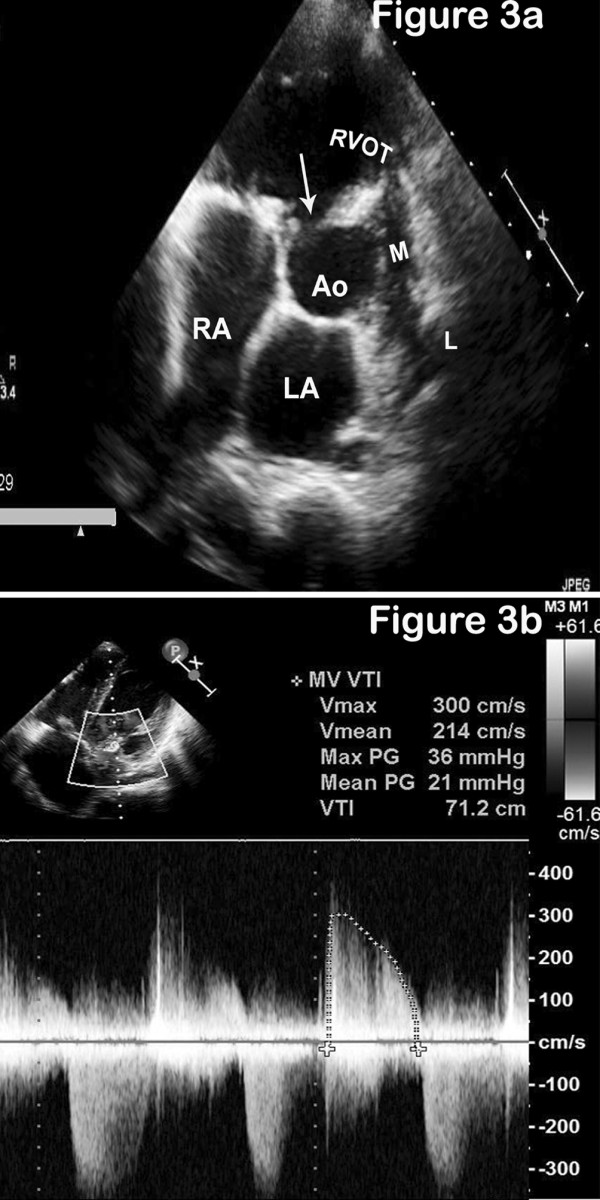
**RVOT morphology and Doppler study of the mitral valve.** (A) Short axis image demonstrating the subaortic ventricular septal defect (arrow), hypoplastic right ventricular outflow tract (RVOT), main pulmonary artery (M) with confluent branch pulmonary arteries. L, left pulmonary artery; RA, right atrium; LA, left atrium. (B) Image demonstrating Doppler gradients across the mitral valve.

An echocardiogram revealed a large malaligned ventricular septal defect with 60% aortic override. The aortic valve was trileaflet with a vegetation on the right coronary cusp (Figure 1). The mitral valve was thickened. Diastolic doming of the anterior leaflet, fixed posterior mitral leaflet with paradoxical motion and two well-formed papillary muscles were noted (Figure 2). There was no aortic stenosis and grade II aortic regurgitation was noted in addition to severe infundibular and annular stenosis with confluent branch pulmonary arteries (Figure 3A). Non calcific severe mitral stenosis with commissural fusion and thickening of the sub-valvular apparatus was noted. The mitral valve area was 1.1 cm^2^. There was no mitral regurgitation. The peak and mean gradients across the valve were 36 and 21 mmHg respectively (Figure 3B) and the echocardiographic mitral valve score was 6/16. Blood cultures revealed Streptococcus viridans as the infecting organism. The serum antistreptolysin O titers were within reference range, C-reactive protein was positive and erythrocyte sedimentation rate was elevated.

A final diagnosis of TF, subacute bacterial endocarditis of the aortic valve and severe mitral stenosis, probably of rheumatic etiology, was considered. Endocarditis with aortic regurgitation added to the hemodynamic burden and the patient succumbed to infective complications during the course of stabilization.

## Conclusion

Although there was no definitive evidence of prior streptococcal infection, the clinical profile and the echocardiographic findings suggest an acquired rheumatic etiology in our patient. Congenital mitral stenosis can present with some leaflet thickening and commissural fusion; however, its association with TF is extremely rare [8]. Fifty percent of symptomatic infants with isolated congenital mitral stenosis are known to die within the first six months of life [5]. The late survival of our patient in the presence of a major associated intracardiac lesion makes congenital pathology unlikely.

About 50% of patients with rheumatic heart disease may not have a prior history of rheumatic fever [9] and recurrent subclinical or unsuspected active carditis leading to late mitral stenosis may occur in the natural history. Histologic evidence of active rheumatic carditis (noted in up to 40% of patients with unexplained heart failure), raised antistreptolysin O titers and absence of other features of carditis support this contention [10]. These arguments favor a rheumatic etiology for the mitral stenosis in our case.

This report draws attention to an interesting association of rheumatic mitral stenosis in TF and highlights the possibility of a coexistent rheumatic lesion in patients with congenital heart disease.

## Competing interests

The authors declare that they have no competing interests.

## Authors' contributions

GVR, NKP and MD carried out the diagnostic evaluation and stabilization. CSK was responsible for drafting the manuscript, its revision and preparation of illustrations. Final edits were carried out by NKP. All the authors read and approved the final manuscript.

## Consent

Written informed consent was obtained from the patient's next-of-kin for publication of this case report and the accompanying images. A copy of the written consent is available for review by the Editor-in-Chief of this journal.
